# Intoxication of Host Cells by the T3SS Phospholipase ExoU: PI(4,5)P_2_-Associated, Cytoskeletal Collapse and Late Phase Membrane Blebbing

**DOI:** 10.1371/journal.pone.0103127

**Published:** 2014-07-25

**Authors:** Hiromi Sato, Dara W. Frank

**Affiliations:** Center for Infectious Disease Research, Department of Microbiology and Molecular Genetics, Medical College of Wisconsin, Milwaukee, Wisconsin, United States of America; Texas A&M Health Science Center, United States of America

## Abstract

*Pseudomonas aeruginosa* is an opportunistic pathogen that is associated with hospital-acquired infections, ventilator-associated pneumonia, and morbidity of immunocompromised individuals. A subpopulation of *P. aeruginosa* encodes a protein, ExoU, which exhibits acute cytotoxicity. Toxicity is directly related to the phospholipase A_2_ activity of the protein after injection into the host cytoplasm via a type III secretion system. ExoU enzymatic activity requires eukaryotic cofactors, ubiquitin or ubiquitin-modified proteins. When administered extracellularly, ExoU is unable to intoxicate epithelial cells in culture, even in the presence of the cofactor. Injection or transfection of ExoU is necessary to observe the acute cytotoxic response. Biochemical approaches indicate that ExoU possesses high affinity to a multifunctional phosphoinositide, phosphatidylinositol 4,5-bisphosphate or PI(4,5)P_2_ and that it is capable of utilizing this phospholipid as a substrate. In eukaryotic cells, PI(4,5)P_2_ is mainly located in the cytoplasmic side of the plasma membrane and anchors adaptor proteins that are involved in cytoskeletal structures, focal adhesions, and plasma membranes. Time-lapse fluorescent microscopy analyses of infected live cells demonstrate that ExoU intoxication correlates with intracellular damage in the early phases of infection, such as disruption of focal adhesions, cytoskeletal collapse, actin depolymerization, and cell rounding. At later time points, a membrane blebbing phenotype was prominent prior to the loss of the plasma membrane integrity and barrier function. Membrane blebbing appears to accelerate membrane rupture and the release of intracellular markers. Our data suggest that in eukaryotic host cells, intracellular ExoU targets and hydrolyzes PI(4,5)P_2_ on the plasma membrane, causing a subsequent disruption of cellular structures and membrane integrity.

## Introduction

*Pseudomonas aeruginosa* is a Gram-negative opportunistic pathogen and a causative agent for hospital-acquired infection, ventilator-associated pneumonia (VAP), and infection in immunocompromised individuals [1]–[3] including patients with burns, AIDS, neutropenia, leukemia, cystic fibrosis, or transplantation patients treated with immunosuppressive therapy [4]–[9]. Mortality rates can be high among *P. aeruginosa*-infected VAP, cystic fibrosis, cancer, and burn patients due to difficulties in treatment because of intrinsic and acquired resistance to antibiotics [6], [8], [10], [11].

*P. aeruginosa* virulence is highly correlated to the expression of a specialized type III secretion system (T3SS) [12], [13]. This system is responsible for intoxicating host cells with up to four effector proteins; ExoS, ExoT, ExoY and ExoU [14]–[20]. In clinical settings, ExoU is a marker for highly virulent strains and has been observed to cause necrotic cell death *in vitro* and *in vivo* [20]–[25]. ExoU possesses a potent phospholipase A_2_ (PLA_2_) activity that requires the participation of an intracellular eukaryotic cofactor [26]. The identified cofactors for ExoU are ubiquitin and ubiquitinated proteins [27], [28]. The eukaryotic specificity of the cofactors allows *P. aeruginosa* to selectively intoxicate host cells without affecting the bacterium producing this toxin. The toxicity of ExoU has been studied in numerous host systems, such as yeast, amoeba, mammalian tissue cultured cells (epithelial and macrophage), and mice [20], [26], [29]–[32]. In animal models, ExoU expression induces lung injury and sepsis [22], [31].

After delivery by the type III injectisome or expression within a eukaryotic host cell, it is believed that ExoU requires activation through the interaction of its carboxyl-terminal domain with the cofactor ubiquitin and plasma membrane phospholipid substrates to result in cytotoxicity [33]–[37]. Among membrane lipids, ExoU possesses high affinity for phosphatidylinositol 4,5-bisphosphate or PI(4,5)P_2_ [36]. The amount of this glycophospholipid relative to total membrane phospholipids is low (0.23–1.4%) and this molecule mostly resides at the inner leaflet of the plasma membrane [38]–[41]. PI(4,5)P_2_ is a biologically important phospholipid in eukaryotic cells and is involved in signaling pathways governing cytoskeletal organization and dynamics, cell adhesion and motility, and membrane trafficking [38], [40], [42]–[44]. PI(4,5)P_2_ controls membrane adhesion by modulating local interactions between the plasma membrane and cytoskeletal components [42],[43]. Also, PI(4,5)P_2_ directly binds to focal adhesion molecules (e.g. talin and vinculin) and other adaptor proteins, playing a critical role in cell-matrix and cell-cell adhesion [43]–[45]. Modulation of this phosphoinositide causes membrane blebbing, changes in focal adhesion and the actin cytoskeleton in mammalian cells [46], [47].

We previously utilized *Saccharomyces cerevisiae* as a model system to correlate PLA_2_ activity and ExoU-mediated toxicity [26]. In these studies we documented the effect of ExoU expression upon intracellular organelles, in which the most prominent phenotype was vacuole fragmentation [26], [48]. The actual mechanism of cellular death due to ExoU, however, is not fully understood. In this study, we focused on analyses to highlight the biological events of host cells after ExoU translocation. Our live-imaging analyses of infected cells demonstrate significant intracellular changes, such as the disruption of focal adhesion, cytoskeletal collapse, and membrane blebbing prior to the loss of plasma membrane integrity. These events appear to correlate with the high affinity interaction and cleavage of the biologically important, multifunctional PI(4,5)P_2_.

## Results

### ExoU translocation and cytotoxicity as assessed in human epithelial cells

**Figure 1:**
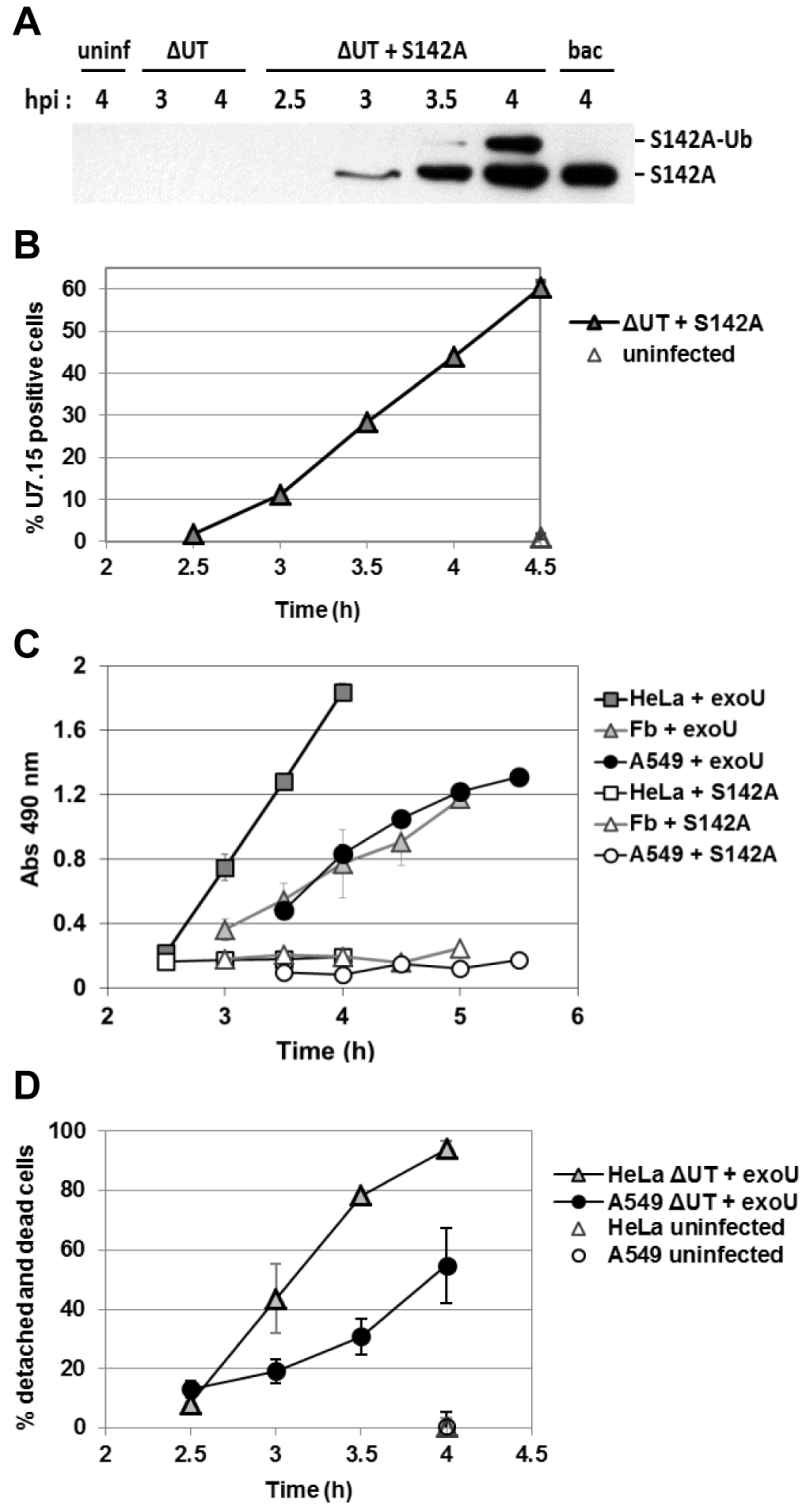
T3S-mediated translocation of ExoU-S142A and ExoU cytotoxicity in human epithelial cells. **A**) After HeLa cell infection (at an MOI of 2.5 throughout Fig. 1), translocated ExoU-S142A was detected by Western blot analysis. The higher MW band is the ubiquitinated form of ExoU-S142A. uninf: uninfected, PA103ΔUT (ΔUT), PA103ΔUT + *exoU-S142A* (ΔUT + S142A), PA103ΔUT + *exoU-S142A* alone (bac). **B**) The percentages of ExoU-S142A-positive HeLa cells during infection quantified by flow cytometry. Cells were infected with PA103ΔUT + *exoU-S142A*, harvested at the indicated time points, and labeled with the anti-ExoU antibody U7.15 and Alexa Fluor 488-conjugated secondary antibody. **C**) LDH release from HeLa, A549, and fibroblast (Fb) cells representing the late stage of toxicity. Cells were infected with PA103ΔUT + *exoU* or *exoU-S142A* and released LDH was measured using a 6-well format at the indicated time points. **D**) The total percentage of intoxicated cells that includes detached cells from the culture plate and dead cells were quantified by flow cytometric analysis after staining with propidium iodide.

During infection, ExoU is delivered into host cells through the T3S injectisome. Due to the acute and potent toxic effect of ExoU, the wild-type protein delivered into epithelial or yeast cells is undetectable in either infected- or transfected-cellular models [26], [49]. To characterize the translocation pattern of this protein, we examined the timing and delivery of non-cytotoxic ExoU-S142A into HeLa cells during infection at a multiplicity of infection (MOI) of 2.5 (the same MOI was used for experiments throughout Fig 1). Strains were designed to eliminate the contribution of ExoT so that the biological effects of ExoU could be examined exclusively (described in Materials and Methods). The T3S-injection rate was biochemically determined by detecting the change in ExoUS142A levels over time after lysing infected host cells with 0.05% Triton. Control experiments indicate that *P. aeruginosa* does not release intracellular markers or lyse in the presence of 0.05% Triton (not shown). Injected ExoU-S142A was detectable at 3 h post-infection (hpi). The translocation rate or intracellular toxin accumulation readily increased after 3.5 h (Fig 1A). Bacterial contamination was undetectable in host cell fractions as assessed by using a highly sensitive anti-LPS antibody (not shown). Two distinct bands, migrating at two molecular masses, represent unmodified ExoU-S142A and ubiquitinated protein (Fig 1A, [50]).

To assess the cell population in which ExoU-S142A is delivered by the T3SS, infected HeLa cells were labeled with an anti-ExoU antibody and an Alexa Fluor-conjugated secondary antibody, and analyzed by flow cytometry. The percentage of ExoU-S142A positive cells was 10% of total cell population at 3 hpi and the increase was prominent after 3.5 hpi (Fig 1B) supporting the apparent translocation rate measured in biochemical experiments (Fig 1A).

ExoU-mediated cell lysis was measured by the release of lactate dehydrogenase (LDH) from cells with compromised plasma membranes. Human epithelial cells including HeLa (cervix), fibroblast (foreskin), and A549 (lung) cells were infected with *P. aeruginosa* strains expressing a cytotoxic (*exoU*) or non-cytotoxic (*exoU*-*S142A*) allele of ExoU. In this experimental model the bacteria express ExoU as the sole effector protein (described in Materials and Methods). The overall pattern of LDH release was analogous among the cell lines tested, the timing of cell lysis, however, appeared to vary (Fig 1C). At 3.5 hpi, 20% of HeLa cells, 15% of fibroblasts, and 10% of A549 cells released LDH using detergent-lysed cells as a maximal reference (not shown), suggesting a trend that killing of HeLa cells was slightly faster than fibroblasts or A549 cells (not statistically significant). Infection with a strain expressing the non-catalytic allele did not release LDH even after longer incubation times (5.5 h, *p*<0.001).

HeLa cells were also subjected to a cytotoxicity test measuring an early stage of cell death. A cell-impermeant small nucleic acid dye, propidium iodide (668 Da), labels cells with compromised plasma membranes. The permeabilization of this nucleic acid dye requires only slight membrane damage compared to the influx of fluorescently-labeled 10 kDa-dextran (not shown) or the release of LDH (140 kDa), which are indicative of prominent membrane damage and cell lysis. When confluent adherent epithelial cells were intoxicated with ExoU, the cells rounded and detached from the plastic substrate, similar to the phenotype of cells exposed to the Rho-GAP domain of ExoS or ExoT [18], [19]. Microscopy-based assays used to quantify intoxicated cell numbers did not include detached cells assessed in these analyses (not shown). We hypothesized that the actual number of cells affected by ExoU requires a method to detect dead cells that are still attached to the culture plate and cells that have detached from the substrate. The number of infected cells stained with propidium iodide and detached from the culture dish was quantified by flow cytometry. At 3.5 hpi, 78% of the total number of HeLa cells were detached or stained with the dye (Fig 1D). The number of affected A549 cells (31%) was lower than HeLa cells (not statistically significant, Fig 1D), recapitulating the cell type-specific trend observed in the LDH release data (Fig 1C).

Overall these data indicate that the detection of the total number of cells intoxicated and killed by ExoU reflects the intoxication events during both early and late stages of infection. We concluded that the rounded cells may represent a population that is affected by the biological action of ExoU prior to cell death.

### ExoU-induced host cell rounding and plasma membrane blebbing prior to cell death

**Figure 2:**
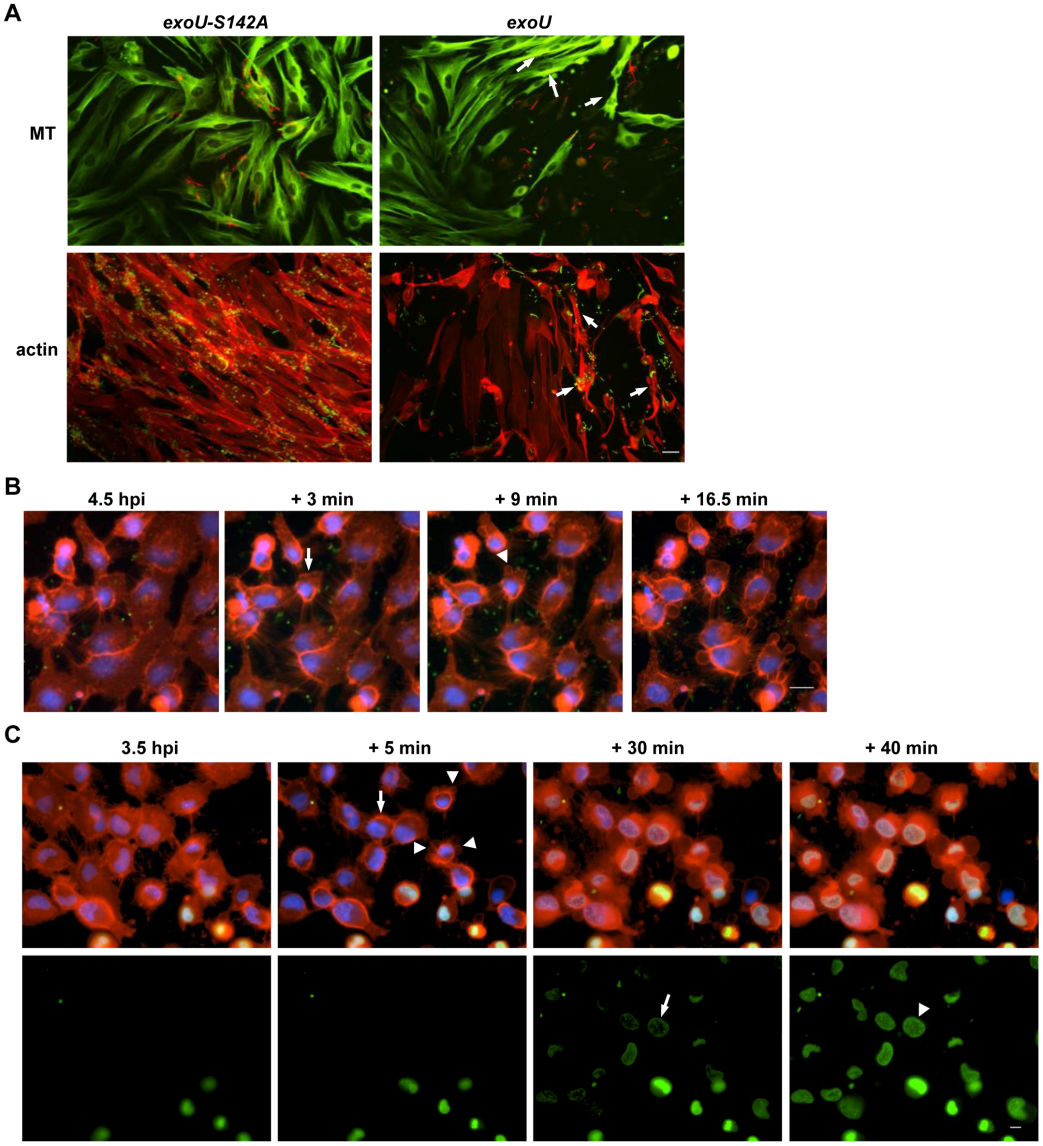
ExoU-induced host cell rounding and plasma membrane blebbing prior to cell death. **A**) Cytoskeletal changes and membrane perturbation are induced before cell death. Fibroblasts were infected with PA103ΔUT + *exoU-S142A* or + *exoU*. Top panels: Microtubules (MT) and *P. aeruginosa* were immunolabeled with an anti-tubulin antibody (green) and an anti-LPS antibody (red), respectively. Bottom panels: The actin cytoskeleton was stained with phalloidin (red) and labeled PA103 is shown in green. ExoU-mediated cytoskeletal disruption is indicated with arrows. Scale bar: 25 µm. Data shown are represenative of 6 experiments. **B**) ExoU-induced morphological changes in host cells during infection analyzed by time-lapse microscopy. HeLa cells were infected with a *P. aeruginosa* strain expressing both GFP and ExoU at an MOI of 2.5. The CellMask plasma membrane stain (red) and permeant Hoechst 33342 (blue) were added 20 min before the start of imaging. Cell rounding is marked with an arrow (+ 3 min) and membrane blebbing is marked with an arrowhead (+ 9 min). The acquired images were cropped (scale bar: 20 µm). Data shown are represenative of 6 experiments. **C**) The timing of membrane blebbing compared to the onset of plasma membrane damage. Cells were infected with an ExoU-expressing strain at an MOI of 5, incubated in the presence of the dyes as used for Fig 2B. The timing of plasma membrane damage was determined as SYTOX green influx, which is shown in bottom panels as corresponding to the multichroic images (top panels). Cell rounding and membrane blebbing are indicated with an arrow and arrowheads, respectively (in the +5 min, multichroic panel). The influx of SYTOX green stained from the edge of the nucleus (an arrow in the +30 min panel) progressing towards the center of the nucleus over time (an arrowhead in the +40 min panel). Scale bar: 10 µm. Data shown are represenative of 21 experiments.

When epithelial cells were infected with an ExoU-producing strain, adherent cells rounded and detached from the culture dish substrate (Fig 1D). To examine this phenomenon further, human fibroblasts, a cell type expressing prominent cytoskeletal structure, were infected and subjected to immunofluorescence microscopy analysis. In healthy fibroblasts, microtubules and the actin cytoskeleton are linearly arranged in elongated cell bodies as seen in the cells infected with a strain expressing the *exoU-S142A* allele (Fig 2A). In contrast, intoxication with ExoU disrupted cytoskeletal morphology causing immunolabeled microtubules and actin signals to become intense or diffuse (arrows, Fig 2A). *P. aeruginosa* expressing ExoU, labeled with an anti-LPS antibody, localized to the most affected areas of the monolayer (Fig 2A).

Having speculated that cell rounding is an early event in ExoU-mediated intoxication, we wondered if the timing of metabolic death was different from the onset of cell rounding. To test this hypothesis, we examined the mitochondrial health and metabolic activity of infected fibroblasts by measuring their oxidative activity using a mitochondria-specific probe, MitoTracker CM-H_2_Xros. After a 4 h infection at an MOI of 2.5, cells infected with an ExoU-expressing strain that remained in contact with the substrate, including the rounded cells, were actively respiring (not shown). The signal of this probe became diffuse or was lost upon cell lysis. In addition, rounded cells appeared alive and metabolically active based on the reduction of MTT by cytosolic NAD(P)H-dependent oxidoreductase enzymes (not shown). These data suggest that morphologically rounded cells are alive, metabolically active and not in a resting state or dead until later time points during infection.

To analyze or characterize the timing of cell rounding, HeLa cells were infected with a strain expressing GFP and ExoU (PA103 *exoT::Tc* + *gfp*) to follow the interaction of bacterial and host cells relative to the cell rounding phenotype. The CellMask plasma membrane dye and nuclear stain Hoechst 33342 were added prior to time-lapse imaging. Bacterial cells transiently interacted with host cells (Movie S1). During these experiments, we noted that ExoU translocation resulted in cell rounding first followed by membrane blebbing at a later time point (Fig 2B, Movie S1). Time-lapse microscopy was an indispensable methodology to observe these dynamic phenotypes (Fig 2B, Movie S1).

To determine the timing of membrane blebbing relative to the onset of plasma membrane damage, the influx of impermeant SYTOX green dye into HeLa cells was examined during infection with an ExoU-expressing strain (PA103 *exoT::Tc*). The first morphological change was cell rounding followed by plasma membrane blebbing (+5 min in Fig 2C, Movie S2). At this point, rounded and cells exhibiting membrane blebs appeared intact by a general absence of SYTOX dye influx. After 30 min of time-lapse imaging (4 hpi), the membrane blebs became enlarged and SYTOX gradually moved into the cell, staining from the edge of the nucleus towards the center of the nucleus over time (+30 min and +40 min in Fig 2C, Movie S2). Nuclear shrinkage was observed in some cells but not in all rounded cells (Table 1). The dynamic movement of blebs stopped as soon as the plasma membrane was compromised at later time points. These results indicate that the cell rounding and plasma membrane blebbing phenomena occur prior to membrane rupture and cell death.

**Table 1:** Biological characteristics of ExoU-mediated toxicity to HeLa cells compared to other cell death mechanisms.

		cell	nuclear	membrane	
	toxicity	rounding	shrinkage	blebbing	blebs
**ExoU delivered by PA103 T3SS**	++	+++	+/□	+++	various sizes, shapes, tubules
**Apoptosis**	+	+++	+++	++	small
**Surfactant**	+++	□	□	□	□
**lyso-PC**	++	□	□	□	□
**honeybee PLA_2_ (675 pmol)**	++	+/□	+/□	+/□	□
**rExoU + Ub** *	□	□	□	□	□
**rExoU + Ub + PI(4,5)P_2_** †	□	□	□	□	□

*Cells were incubated with 1.35 to 675.65 pmol of rExoU in the presence of penta-ubiquitin (13.51 to 675.65 pmol) as the cofactor.

† Cells were incubated with 67.57 pmol of rExoU in the presence of penta-ubiquitin (13.51 or 65.57 pmol) and 5 nmol PI(4,5)P_2_.

### Phenotypes of ExoU-mediated cytotoxicity compared to other cell death mechanisms

**Figure 3:**
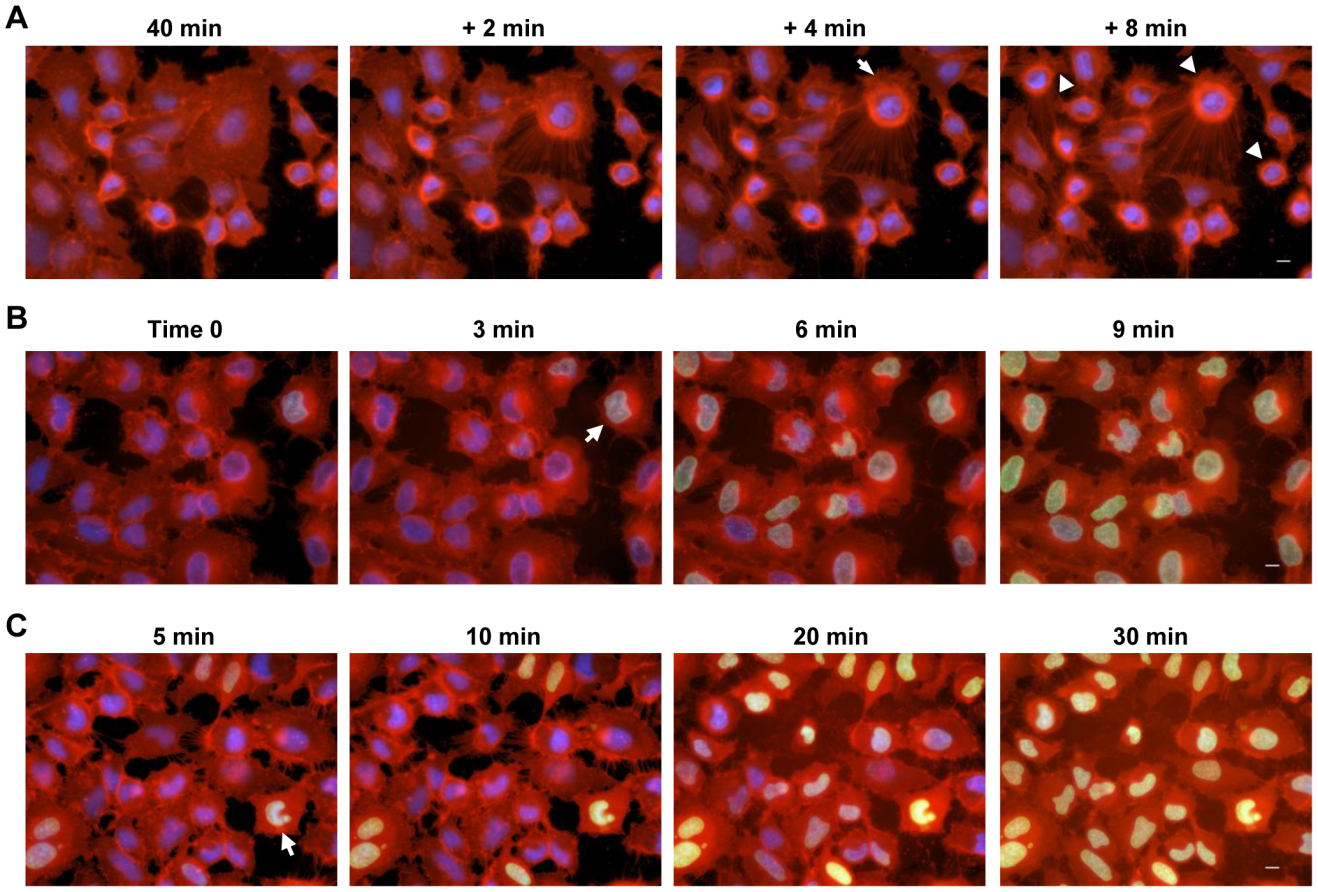
Phenotypes of cell death caused by apoptosis, surfactant application, and honeybee PLA_2_. **A**) Apoptotic, programmed cell death of HeLa cells induced by incubation with 3 µM gliotoxin. Apoptotic membrane blebbing was recorded by time-lapse microscopy for measurement analyses. Cell rounding (an arrow in the +4 min panel) and apoptotic blebbing (arrowheads in the +8 min panel) are highlighted. Cells were stained as Fig 2D. Scale bar: 10 µm. Data shown are represenative of 3 experiments. **B**) Cell death caused by surfactant-based membrane rupture. To image the immediate action of the detergent, 100 µl of 0.1% Triton X-100 solution was gently dropped into the culture dish and the reaction of HeLa cells was followed by time-lapse imaging. Within 3 min, SYTOX green crossed the plasma membrane (an arrow in the +3 min panel). Scale bar: 10 µm. Data shown are represenative of 5 experiments. **C**) Intoxication of HeLa cells caused by the addition of 675 pmol honeybee venom PLA_2_ to the dish. The plasma membrane was compromised within 5 min, as indicated by an arrow. Scale bar: 10 µm. Data shown are represenative of 5 experiments.

In general, the phenotypes of cell rounding, nuclear shrinkage, and membrane blebbing are the hallmarks of apoptotic, programmed cell death [51]. This led us to evaluate the phenotypes of cell death induced by other causes, compared to the phenotypes of ExoU-mediated intoxication and cell death. The characteristics of apoptotic cell death induced by gliotoxin were confirmed by time-lapse microscopy as nuclear condensation, cell rounding, and plasma membrane blebbing (Fig 3A, Movie S3, summarized in Table 1). Apoptotic cell nuclei were tightly condensed, cell rounding was compact in size, and the shape of blebs was uniform and small in size (Fig 3A, Movie S3). In apoptosis, the average diameter of blebs was 2.02 µm±0.87 µm (Table S1). In contrast, during ExoU intoxication, we observed multiform blebs of various sizes as well as plasma membrane tubules. The diameter of ExoU-mediated blebs was longer, ranging from 1.87 to 16.94 µm (average of 7.82 µm±2.06 µm), and the tubule length ranged between 11.16 and 30.93 µm (Table S1). Annexin V binds to apoptotic plasma membranes but did not bind to ExoU-intoxicated HeLa cells (not shown). In contrast to the circular shape of apoptotic cells, the shape of ExoU-intoxicated cells varied and appeared not as well controlled as during apoptotic death. These results are supported by Kaufman *et al.* who demonstrated that PA103 infection caused classical necrotic morphology in the absence of DNA fragmentation or Caspase-3 activity [52].

Having PLA_2_ activity, ExoU is believed to localize to and degrade the plasma membrane [33], [34], [50]. The products of the PLA_2_ enzymatic activity include lyso-phospholipids, which are natural surfactants that disrupt lipid-lipid, lipid-protein and protein-protein interactions in the plasma membrane. To examine the action of surfactant-based membrane rupture, we first tested a synthetic, nonionic detergent Triton X-100. The surfactant solution immediately caused damage to plasma membranes as observed by diffusion of the membrane staining pattern and a morphology change in the edge of cells (Fig 3B, Movie S4). Within 3 min, the impermeable SYTOX green dye crossed the plasma membrane and stained the nucleus, indicating cell death. Cell rounding, nuclear shrinkage, and membrane blebbing phenotypes were undetectable (Movie S4, Table 1).

The extracellular addition of a lyso-phospholipid, lyso-PC (1-palmitoyl-2-hydroxy-sn-glycero-3-phosphocholine), exhibited subtle cell shrinkage rather than rounding (Movie S5). The cell shrinkage occurred in each cell in an independent manner rather than the pulling action on adjacent cells that we noted in ExoU-mediated intoxication. As soon as the plasma membrane was ruptured by lyso-PC, the plasma membrane dye flowed in and the cell and nucleus swelled, which is a hallmark of necrosis (Movie S5, Table 1).

We then evaluated cell death induced by extracellular PLA_2_. Honeybee (*Apis mellifer*) venom PLA_2_ was gently added to the HeLa cell monolayer for time-lapse imaging analysis. The action of this extracellular PLA_2_ caused minor amounts of cell rounding, nuclear shrinkage, or membrane blebbing (Fig 3C, Movie S6, Table 1). The plasma membrane was compromised within 5 min of the honeybee PLA_2_ addition (Fig 3C).

Finck-Barbancon *et al*. and Phillips *et al.* demonstrated that purified ExoU alone is not cytotoxic when applied to the surface of CHO cells [20], [33]. In this study, we added equal molar concentrations of the cofactor ubiquitin to determine whether activation of rExoU would result in cytotoxicity. Based on microscopy analysis of propidium iodide-stained cells, extracellular rExoU with ubiquitin did not intoxicate HeLa cells even after an overnight incubation (Table 1). After 1 to 4 h of incubation with HeLa cells, rExoU with ubiquitin retained 74% or more of PLA_2_ activity and ∼60% activity after 22 h incubation (not shown). Based on Western blotting analyses, the amounts of rExoU were similar during the first 4 h of incubation or after overnight incubation (not shown). We then tested the extracellular effect of native ExoU released from PA103Δ*pcrV*, a deletion strain of the T3S translocator gene *pcrV*. This deletion strain is unable to deliver any type III effector proteins into host cells and is nontoxic *in vitro* and *in vivo* [53]. During infection of mammalian cells, ExoU expressed in this strain is released into culture medium [54]. We infected HeLa cells with PA103Δ*pcrV* and observed that the native form of ExoU that was released in the medium did not intoxicate cultured cells in the presence of added cofactors, recapitulating the rExoU data (Table 1).

We examined the effect of cell contents released from host cells that were killed by infection with PA103ΔUT + *exoU*. The released cell constituents should include injected-ExoU, cellular cofactors for ExoU, ubiquitinated-ExoU, and other cellular components. The cell contents released in culture medium were filter sterilized and extracellularly added to uninfected HeLa cells. Cells were unaffected after overnight incubation (not shown).

Overall, the intoxication mechanism of injected ExoU was different from apoptotic cell death, surfactant-based membrane rupture, extracellularly-added honeybee PLA_2_, or a product of PLA_2_ activity, lyso-phospholipids. Extracellular ExoU did not damage host cells even in the presence of its cofactor.

### Effect of PI(4,5)P_2_ on ExoU-mediated cytotoxicity in eukaryotic host cells

Immunofluorescence microscopy analyses indicated that ExoU appeared to alter the cytoskeleton and focal adhesion of mammalian epithelial cells (Fig 2A). In addition, Gendrin *et al*. demonstrated the selective binding of rExoU to PI(4,5)P_2_ [36], which is a biologically important phospholipid in eukaryotes used as an anchor between the cytoskeleton, plasma membrane, and focal adhesions [46], [47]. We postulated that PI(4,5)P_2_ links ExoU intoxication to complex biological phenomena, including cytoskeletal changes and plasma membrane blebbing.

**Figure 4:**
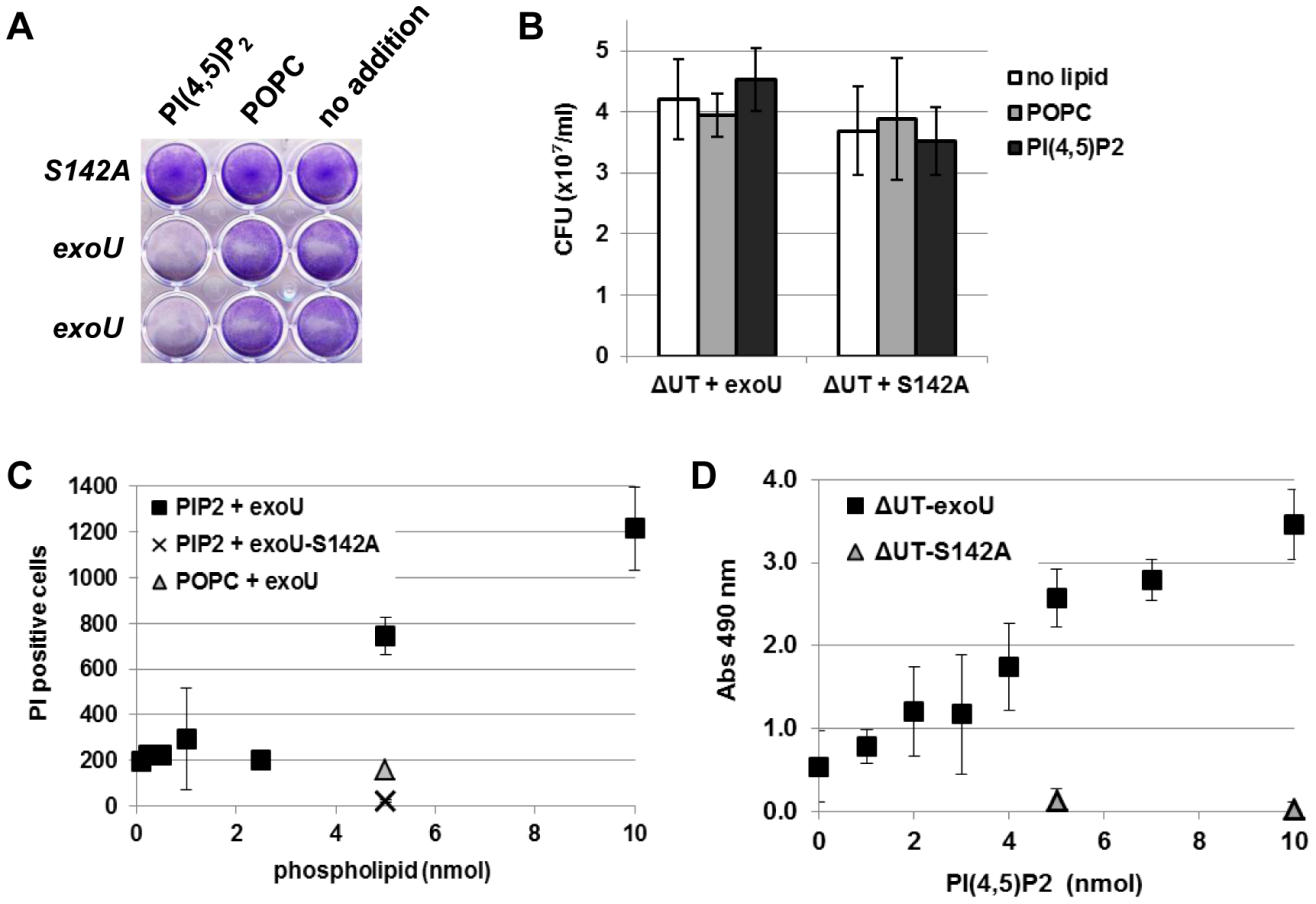
Effect of PI(4,5)P_2_ on ExoU-mediated cytotoxicity. **A**) The addition of PI(4,5)P_2_ increased ExoU-induced cell detachment from a culture plate. HeLa cells were pre-incubated with PI(4,5)P_2_ or POPC or without additional phospholipids (no addition), infected with PA103ΔUT + *exoU* or *exoU-S142A*, and analyzed with a cell retention assay using crystal violet staining. **B**) Bacterial growth in the presence of additional phospholipids during infection. HeLa cells were preincubated with phospholipids for 1 h and infected. After 4 h, the culture medium was collected and subjected to a CFU assay. **C**) Increased efficacy of ExoU cytotoxicity in the presence of PI(4,5)P_2_. HeLa cells were preincubated with the indicated amounts of phospholipids for 1 h, infected at MOI of 1.25 for 4 h to assess the early stage of intoxication. The influx of the impermeant propidium iodide represents cells with the compromised plasma membrane, which were quantified from micrographs of 3 independent experiments. **D**) PI(4,5)P_2_ dose-dependency of ExoU cytotoxicity determined by the LDH release assay. HeLa cells were pre-incubated with indicated amounts of PI(4,5)P_2_ for 1 h, infected at MOI of 2.5 for 4 h to capture the late stages of intoxication and cell lysis, and the release of LDH was measured in a 24-well format.

To test this hypothesis, we examined whether the addition of PI(4,5)P_2_ to cultured cells influences ExoU-mediated cytotoxicity. HeLa cells were pre-incubated with PI(4,5)P_2_ or POPC or without additional phospholipids. POPC is a major component of biological membranes, mostly located in the outer leaflet of the plasma membrane. PLA_2_ activity of rExoU on POPC incorporated in liposomes is highly efficient in the presence of a cofactor [26], [27]. After preincubation, cells were infected for 5 h and the effect of ExoU intoxication was determined by a cell retention assay. The addition of PI(4,5)P_2_ but not POPC increased ExoU-induced cell detachment from culture plates (Fig 4A).

The addition of PI(4,5)P_2_ could augment bacterial replication during infection, which may enhance host cell detachment. To eliminate this as a confounding factor, bacterial growth during cellular infections was evaluated by quantifying colony forming units (CFUs). There was no significant difference in bacterial CFUs either in the presence of PI(4,5)P_2_ or POPC, or in the absence of additional synthetic phospholipids (Fig 4B). These data indicate that the addition of PI(4,5)P_2_ enhanced ExoU-mediated cell detachment through an effect on host cells rather than by a mechanism involving an increase in bacterial growth.

To evaluate the efficacy of ExoU toxicity, HeLa cells were preincubated with various amounts of PI(4,5)P_2_ and then infected with PA103 ΔUT + *exoU* at MOI of 1.25 for 4 h, which represents conditions which can capture events occurring at an early stage of intoxication and membrane damage. The number of membrane damaged cells was quantified by the influx of propidium iodide and fluorescent microscopy analyses. The addition of 5 or 10 nmol PI(4,5)P_2_ significantly increased the number of dead cells in contrast to the amount of 2.5 nmol or less (Fig 4C), suggesting an enhanced cytotoxic effect of ExoU in a dose-dependent manner.

The dose-dependency of PI(4,5)P_2_ was further determined by the LDH release assay. After preincubation with PI(4,5)P_2_, HeLa cells were infected at MOI of 2.5 for 4 h, which are conditions that capture the late stages of intoxication and cell lysis. The magnitude of cell lysis by ExoU related to PI(4,5)P_2_ in a dose-dependent manner (Fig 4D), suggesting the involvement of PI(4,5)P_2_ in the process of ExoU toxicity of eukaryotic cells.

### Enzymatic activity on PI(4,5)P_2_ and high-affinity interaction of ExoU to the specific phospholipid

In the HeLa cell infection model, the addition of PI(4,5)P_2_ to cultured cells considerably increased ExoU toxicity (Fig 4). PI(4,5)P_2_ is a substrate of phospholipase C (PLC) activity, releasing diacylglycerol (DAG) and inositol 1,4,5-triphosphate (IP_3_) as cleavage products [43], [55]. In contrast, only a few members of the PLA_2_ family of enzymes are reported to use PI(4,5)P_2_ as a substrate [56], [57]. We previously characterized the substrate specificity of ExoU PLA_2_ activity *in vitro* using several phospholipids that are main constituents of biological membranes but did not test PI(4,5)P_2_, which resides miniscule amounts in cell membranes [26], [58].

**Figure 5:**
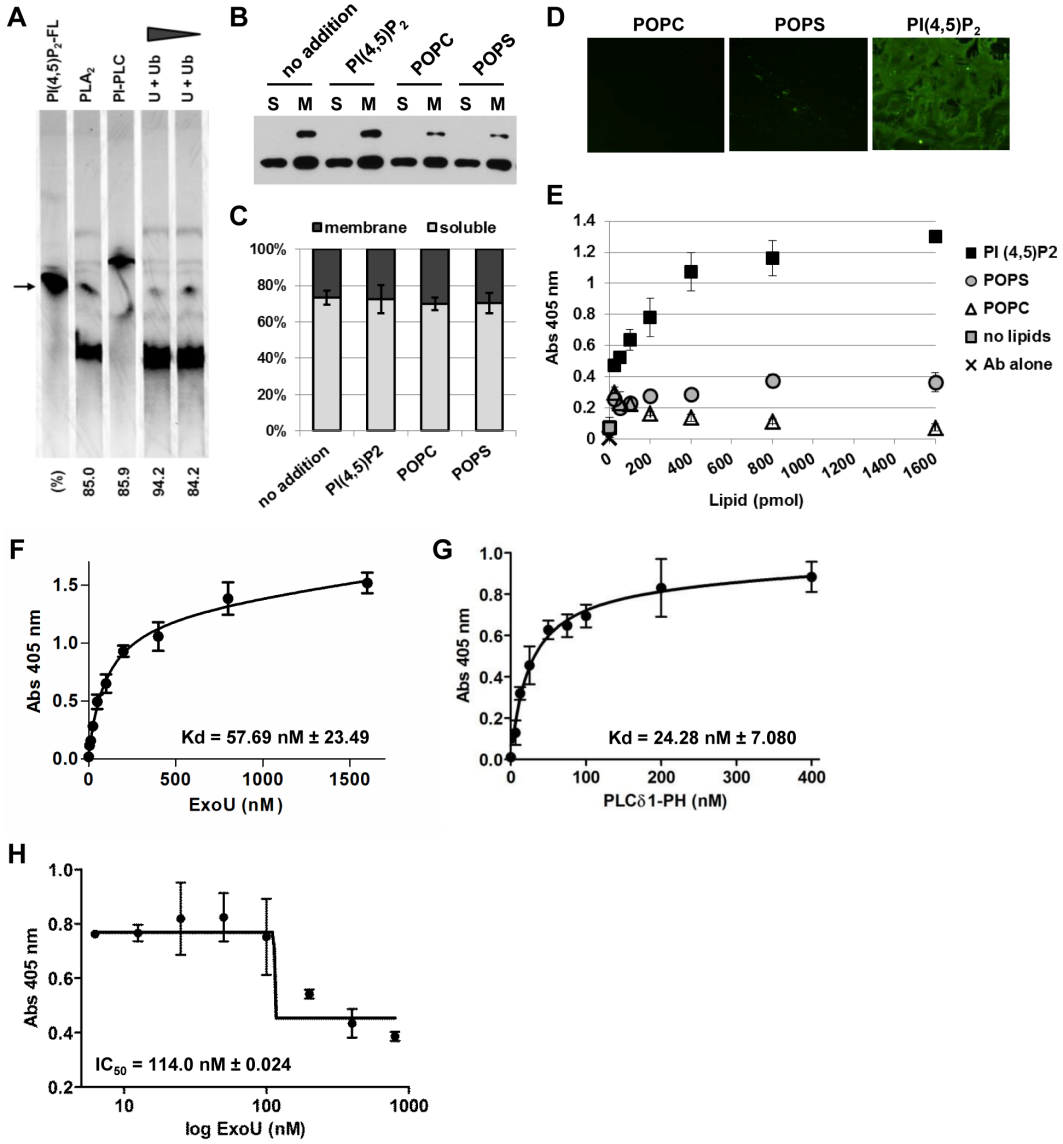
Enzymatic activity on PI(4,5)P_2_ and high-affinity interaction of rExoU. **A**) Enzymatic activity of rExoU on BODIPY-FL-PI(4,5)P_2_ determined by TLC. The fluorescent substrate was incubated with 13.5 or 6.76 pmol rExoU in the presence of the cofactor ubiquitin (13.5 pmol), labeled as U + Ub. PI-PLC and honeybee PLA_2_, 13.5 pmol each, were also tested. The position of the non-hydrolyzed substrate is indicated with an arrow. The percentage hydrolysis of each enzyme, averaged from 3 independent experiments, is shown below the chromatograph. **B**) Localization of ExoU-S142A in HeLa cells after infection in the presence or absence of the extracellular phospholipids. Infected cells were harvested, mechanically lysed, and fractionated. Fractions were analyzed by Western blot using an anti-ExoU monoclonal antibody. S: soluble fraction, M: membrane fraction. To achieve a linear detection range, 4-fold higher volumes of membrane fractions were loaded relative to soluble material. **C**) The Western blot signal intensity of both unmodified and ubiquitinated ExoU-S142A was quantified. Membrane fraction (dark gray) and soluble fraction (light gray) are shown as a 100% stacked column chart with the mean ± SD from 3 independent experiments. **D**) rExoU (13.5 pmol) binding to phospholipid (1600 pmol)-coated polystyrene plates detected by fluorescence microscopy. Magnification: 40x. **E**) rExoU affinity to phospholipids evaluated by an ELISA-based solid-phase binding assay. Polystyrene plates were coated with the indicated amounts of phospholipids. The binding of 13.5 pmol rExoU was determined using an anti-ExoU antibody and a horseradish peroxidase-conjugated secondary antibody. Peroxidase activity on the substrate ABTS with H_2_O_2_ was measured by absorbance at 405 nm. **F**) The Kd of rExoU to PI(4,5)P_2_ determined with the ELISA-based binding assay. Nonlinear regression analysis of the concentration of rExoU (nM) as a function of total binding is shown. The plot represents the mean ± SEM of duplicates from 2 independent experiments. **G**) Binding of the PLCδ1-PH domain to PI(4,5)P_2_ evaluated by nonlinear regression of the concentration of the PLCδ1-PH peptide (nM) as a function of total binding. The plot represents the mean ± SEM of duplicates from 2 independent experiments. **H**) Inhibitory effect of rExoU to PLCδ1-PH binding to PI(4,5)P_2_. rExoU (between 6.25 and 800 nM) was mixed into 50 nM of the PLCδ1-PH peptide for the solid-phase binding assay. IC_50_: the concentration of rExoU to displace 50% of PLCδ1-PH binding. The plot represents the mean ± SEM of duplicates from 2 independent experiments.

To determine if ExoU hydrolyzes PI(4,5)P_2_, the fluorescent substrate, BODIPY-FL-PI(4,5)P_2_ was incubated with rExoU and its cofactor ubiquitin for TLC analysis. ExoU readily cleaved PI(4,5)P_2_, releasing the products, lyso-PI(4,5)P_2_ (BODIPY-FL-labeled, visible in chromatograph) and an unlabeled fatty acid chain (Fig 5A). The migration pattern of the hydrolyzed product of ExoU was identical to that of honeybee PLA_2_ (Fig 5A). Phosphatidylinositol-specific PLC (PI-PLC) was also tested for comparison. In the chromatograph, BODIPY-FL labeled DAG was visible at a higher migration position than the substrate PI(4,5)P_2_ due to the loss of the charged head group in IP_3_ (Fig 5A). These data demonstrate that ExoU hydrolyzes PI(4,5)P_2_ as a substrate, releasing PLA_2_-specific products.

In eukaryotic cells, PI(4,5)P_2_ is located mainly at the inner leaflet of the plasma membranes at low levels. We postulated that the enhancement of ExoU cytotoxicity in the presence of extracellularly added PI(4,5)P_2_ was due to the increased targeting to host cell membranes via this specific substrate. The localization of ExoU-S142A in HeLa cells was analyzed in the presence or absence of the synthetic phospholipids. Cells were subjected to mechanical lysis and differential centrifugation-based fractionation. Western blot analyses demonstrated that the pattern of ExoU-S142A localization in soluble and membrane fractions in the presence of PI(4,5)P_2_ was similar to the pattern in the presence of POPC or palmitoyl-2-oleoyl-sn-glycero-3-phospho-L-serine (POPS) or in the absence of synthetic phospholipids (Fig 5B). Quantification of the chemiluminescent signals indicated no significant difference in the percentage ratio of ExoU-S142A amounts in soluble and membrane fractions for all samples (Fig 5C). Intriguingly, the ubiquitinated protein was detectable in only the membrane fractions under these conditions (Fig 5B).

The addition of PI(4,5)P_2_ had little influence on the amount of ExoU-S142A localized to the membrane fraction (Fig 5C), so how can ExoU intoxicate host cells more efficiently in the presence of this specific phospholipid? Gendrin *et al*. demonstrated the high affinity binding of rExoU to PI(4,5)P_2_ in a lipid-strip assay [36]. We further analyzed the rExoU affinity to PI(4,5)P_2_ and compared these data to other phospholipid substrates. To examine the affinity using a solid-phase binding assay, a constant amount (1600 pmol) of phospholipids was immobilized and the binding of rExoU was detected by fluorescence microscopy using an anti-ExoU monoclonal antibody and an Alexa Fluor-conjugated secondary antibody. Using this non-quantitative assay, it appeared that the binding of rExoU to PI(4,5)P_2_ was significantly higher than the binding to POPS or POPC (Fig 5D).

Next, the binding affinity of rExoU to immobilized phospholipids was quantified by an ELISA-based solid-phase binding assay. The binding of rExoU (13.5 pmol) to PI(4,5)P_2_ was dose-dependent and saturated at 400 pmol or higher amounts of this lipid (Fig 5E). The maximal total binding at equilibrium (Bmax) to PI(4,5)P_2_ was 0.9743+/−0.088. In contrast, the binding affinity of rExoU to POPS was low (Bmax  = 0.2138) and rExoU did not bind stably to POPC (Bmax  = 0.1370). For POPS and POPC, a typical saturation curve was unobtainable.

**Figure 6:**
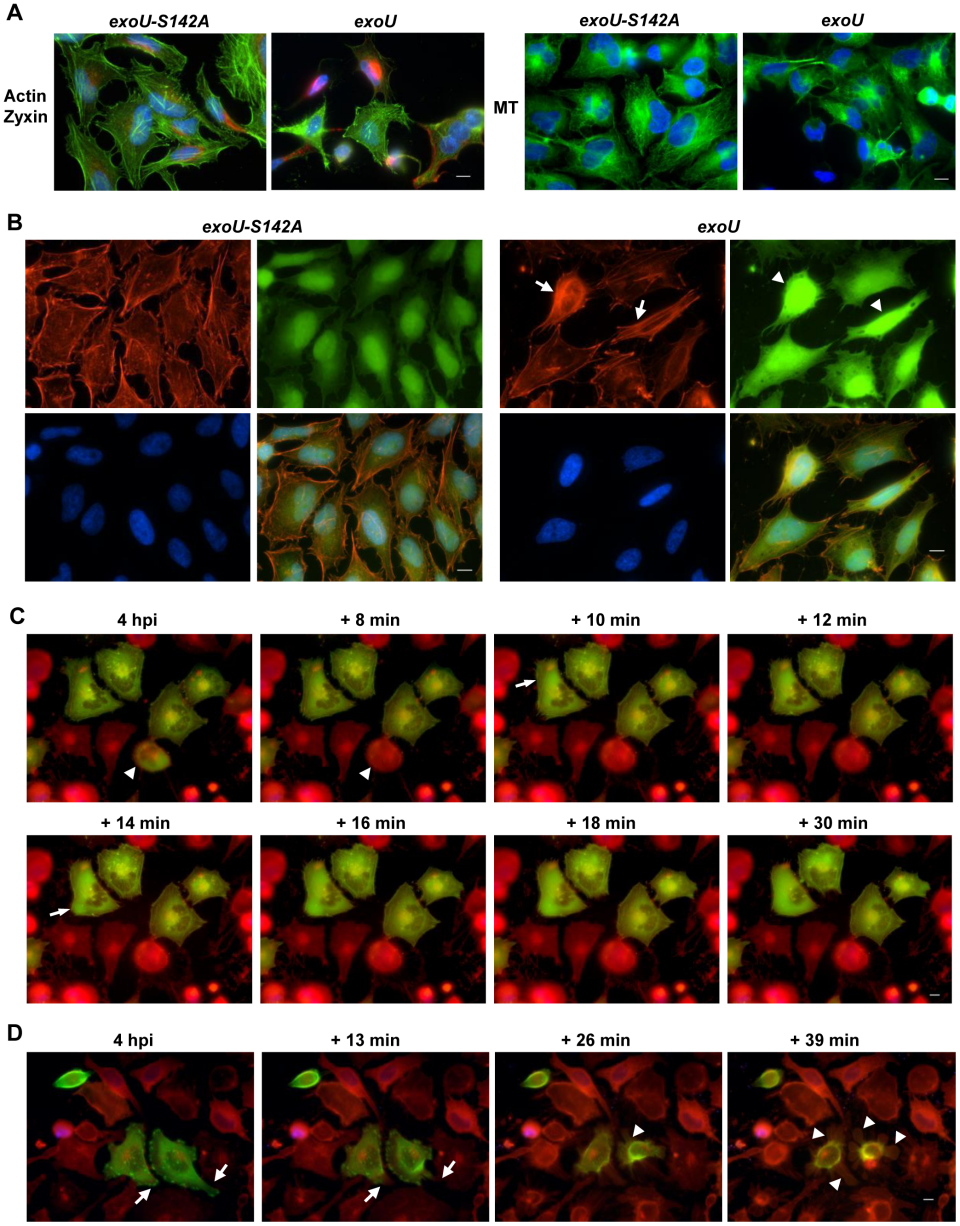
Disruption of focal adhesion as well as actin depolymerization, leading to cytoskeletal collapse. **A**) The effect of ExoU on cytoskeletal structure and focal adhesion patterns in HeLa cells. After infection at MOI of 5 for 3 h 15 min, the actin cytoskeleton and a focal adhesion protein were visualized with phalloidin (green) and an anti-Zyxin antibody (red), respectively. Microtubules (MT) were immunolabeled with an anti-alpha-tubulin antibody (green). DAPI-stained nucleus is shown in blue. Scale bar: 10 µm. Data shown are represenative of 5 experiments. **B**) Depolymerization of actin cytoskeleton caused by ExoU. HeLa cells were infected with PA103ΔUT + *exoU* or *exoU-S142A* at MOI of 5 for 3 h 15 min, fixed, and stained with DNase I (G-actin, shown in green), phalloidin (F-actin, shown in red), and DAPI (blue). ExoU induced the bundled or collapsed morphology of actin filaments (arrows) and actin depolymerization, indicated as an intense G-actin staining pattern (arrowheads). Right bottom panels: Overlay images. Scale bar: 10 µm. Data shown are represenative of 3 experiments. **C**) Live imaging of ExoU-mediated depolymerization of the actin cytoskeleton prior to cell death. HeLa cells expressing GFP-tagged actin were infected and the change in the filamentous actin-GFP was detected by time-lapse microscopy. Cells were also labeled with the CellMask plasma membrane stain (red). The depolymerization of filamentous actin-GFP is highlighted with arrows in the +10 and +14 min panels. Depolymerized actin-GFP molecules were released from the cell upon cell lysis (compare the arrowhead in the 4 hpi panel to the one in the +8 min panel). Scale bar: 10 µm. Data shown are represenative of 10 experiments. **D**) The influence of ExoU on the focal adhesion protein Talin prior to plasma membrane damage. HeLa cells expressing a GFP-fused Talin signal peptide were infected, stained, and analyzed as described in Fig 6C. The GFP-Talin at the edge of cells began detaching from the culture dish and was pulled away from the plasma membrane (compare the arrows in the 4 hpi panel to the +13 min panel). Loss of cell adhesion led to cell rounding and then plasma membrane blebbing (arrowheads in the +26 and +39 min panels). Scale bar: 10 µm. Data shown are represenative of 4 experiments.

Next, the equilibrium-binding constant (Kd) of rExoU to immobilized phospholipids was determined. The Kd of rExoU was 57.69 nM±23.49 for 400 pmol PI(4,5)P_2_ (Fig 6F) and 60.03 nM±31.20 for 200 pmol (not shown) after nonspecific binding of each concentration of rExoU was incorporated in the fit calculation. Thus, the binding affinity of rExoU to PI(4,5)P_2_ was avid, suggesting a biological role for this phospholipid in the intoxication of mammalian host cells.

Among PI(4,5)P_2_-binding proteins, the best characterized is PLCδ1. Its PI(4,5)P_2_ binding motif, PH domain, recruits PLCδ1 to the plasma membrane and is used as a reporter in living cells [42], [47], [59]. In our solid-phase binding assay, the Kd of the purified PLCδ1-PH domain peptide to PI(4,5)P_2_ was 24.28 nM±7.080, approximately half the Kd value of rExoU (Fig 5G).

To assess the competition between rExoU and the PLCδ1-PH peptide for PI(4,5)P_2_ binding, the inhibitory effect of rExoU to PLCδ1-PH binding was determined. When concentrations higher than 100 nM were mixed into 50 nM of PLCδ1-PH, rExoU demonstrated a competitive effect on PLCδ1-PH binding to PI(4,5)P_2_ (Fig 5H). The half maximal inhibitory concentration (IC_50_) of rExoU to displace 50% of PLCδ1-PH binding was 114.0 nM±0.024 (Fig 5H). These data suggest that ExoU may influence the interaction of PI(4,5)P_2_-binding proteins at the plasma membrane and cortical cytoskeleton.

### Disruption of focal adhesion by ExoU PLA_2_ activity, leading to actin depolymerization and cytoskeletal collapse prior to cell death

Injection of ExoU into eukaryotic cells leads to necrotic death ([20]–[25]) but the actual mechanism of killing by ExoU is unknown. In the yeast host system, the vacuole, one of the large yeast organelles, is fragmented upon ExoU expression [26]. We postulated that ExoU might impact intracellular structures, which could contribute to cell death. When mammalian cells were infected, ExoU induced cell rounding and membrane blebbing prior to plasma membrane rupture (Fig 2). Modulation of the cytoskeletal architecture by ExoU (Fig 2) may initiate the cell rounding phenotype or *vice versa*.

We evaluated the effect of ExoU on the cytoskeletal structures and focal adhesion patterns by microscopy using specific probes for each. In the cells infected with the ExoU-expressing strain, the signal of filamentous actin staining became diffuse or intense, or the cell lost typical cytoskeletal architecture or was collapsed (Fig 6A). The signal from focal adhesions, as detected with anti-Zyxin antibody, demonstrated a punctate pattern or became more intense (Fig 6A). Also, tight junction (ZO-1) and cell-cell contacts (vinculin) were disrupted following the delivery of ExoU (not shown). The immunolabeled microtubule fibers appeared woven by braiding or the cytoskeletal structure appeared to collapse (Fig 6A). In summary, the translocation of ExoU, but not ExoU-S142A, damaged focal adhesion and cytoskeletal structures of the host cell.

To analyze the effect of ExoU on the actin cytoskeleton further, the dynamic state of actin proteins was assessed by staining globular actin (G-actin) with DNase I and filamentous actin (F-actin) with phalloidin. In cells infected with PA103ΔUT + *exoU,* the depolymerization of actin filaments was clearly indicated as an intense, diffuse G-actin staining pattern (arrowheads, Fig 6B). The average intensity of G-actin signals in these cells was at least 2- to 3-fold higher than the intensity in cells infected with the nontoxic strain. The actin filaments also changed to a more bundled or collapsed morphology (arrows, Fig 6B), as seen in fibroblasts (Fig 2A).

The progression of the actin cytoskeletal change during infection was analyzed by live imaging. HeLa cells were treated with CellLight, a reagent to visualize the actin cytoskeleton after transduction of a *gfp*-tagged actin gene. After confirming the expression of GFP-fused actin, cells were infected at an MOI of 2.5 and labeled with the CellMask plasma membrane stain and SYTOX blue to determine the timing of the damage to the plasma membrane. After 10 min of time-lapse imaging at 4 hpi, the depolymerization of filamentous actin-GFP was detected accompanied by gradual cell shrinkage (indicated with an arrow in the +10 min panel in Fig 6C, Movie S7). Four min later, actin depolymerization was visualized at another spot within the same cell (an arrow in the +14 min panel in Fig 6C, Movie S7). These data suggest that the depolymerization process occurs as staggered events based on the location of the affected structure in the cell and does not happen all at once. The progression of depolymerizing action was detected prior to the plasma membrane rupture, indicating that the alteration of the actin cytoskeleton was ExoU-specific and not a secondary effect of cell death (Fig 6C, Movie S7). The depolymerized actin-GFP molecules were released from the cell upon membrane rupture (compare the arrowhead in the 4 hpi panel to the one in the +8 min panel in Fig 6C, Movie S7). These data indicate that ExoU mediates the depolymerization of the actin cytoskeleton, leading to cytoskeletal collapse prior to cell death.

Just before starting the actin depolymerization process, the ability of cells to adhere to the culture dish weakened and cells began to round and detach (Movie S7). In Fig 6A, we demonstrated the modulation of the focal adhesion protein Zyxin by ExoU. The dynamics of cell adhesion during infection was evaluated by time-lapse microscopy using the CellLight system to visualize a focal adhesion protein, Talin. A GFP-fused signal peptide for Talin targets the subcellular assemblies of focal adhesions, through which the cytoskeleton of cells adhere to the extracellular matrix. After several min of imaging at 4 hpi, the GFP-Talin located at the edge of cells began detaching from the culture dish substrate and was pulled away from the plasma membrane (arrows in the +13 min panel in Fig 6D, Movie S8). Loss of cell adhesion to the extracellular matrix led to cell rounding and then plasma membrane blebbing (an arrowhead in the +26 min panel in Fig 6D, Movie S8). The blebbing phenomenon became more dynamic over time, as the size of blebs enlarged, and then cells lysed (arrowheads in the +39 min panel in Fig 6D, Movie S8). Thus, ExoU disrupted the focal adhesion, which could damage the connection between the cytoskeleton and extracellular matrix, leading to cell rounding and membrane blebbing prior to plasma membrane rupture.

In summary, these cellular-modification phenotypes can be explained by the capabilities of ExoU to strongly interact with PI(4,5)P_2_ and hydrolyze this biologically important phospholipid that forms the inter-connections between the cortical cytoskeleton, focal adhesion, and plasma membrane sites.

## Discussion

### ExoU has high affinity to PI(4,5)P_2_ and utilizes it as a PLA_2_ substrate

ExoU is an acute toxin delivered into eukaryotic host cells by *P. aeruginosa* T3SS. The PLA_2_ activity of this toxin is required for cytotoxicity. As an enzyme, ExoU targets a broad range of substrates that include synthetic PC, phosphatidylethanolamines (PE), phosphatidic acid (PA), lyso-PC, and also neutral lipids in yeast [26], [58], [60]. PC and PS are major components of the outer leaflet and inner leaflet of the plasma membrane in eukaryotic cells, respectively. The binding affinity of ExoU to anionic PS or cationic PC is, however, extremely low and the dissociation constants for these phospholipids are not measurable under our experimental conditions (Fig 5E).

In this study, we demonstrate that ExoU hydrolyzes PI(4,5)P_2_ as a PLA_2_ substrate (Fig 5A). ExoU also possesses a high binding affinity specifically to PI(4,5)P_2_ (Kd = 57.69 nM, Fig 5E and F, and [36]). Most of known PI(4,5)P_2_-interacting actin-binding proteins interact with this phospholipid at lower affinity. For example, the Kd values of villin and gelsolin are 39.5 µM and 40.2 µM, respectively [61], [62]. The best characterized PI(4,5)P_2_-binding protein PLC-δ1 possesses the specific PH binding-domain and the Kd of this domain is ∼1 µM [40]. In our solid-phase binding assay, the Kd of PLC-δ1-PH domain was 24.28 nM, approximately half of the Kd measured for ExoU (Fig 5G).

In a HeLa cell infection model, we observed that the addition of exogenous PI(4,5)P_2_ increases ExoU-mediated cytotoxicity as compared to conditions where there is no or POPC addition (Fig 4). One possibility is that exogenous PI(4,5)P_2_ incorporates in the plasma membrane and provides an additional high affinity substrate. Tyson and Hauser demonstrated that PI(4,5)P_2_ increases ExoU PLA_2_ catalytic activity on an immobilized PC substrate in their *in vitro* assay [63]. It is possible that ExoU utilizes PI(4,5)P_2_ as a scaffold or an adaptor to engage the plasma membrane and hydrolyze neighboring lipid substrates in addition to PI(4,5)P_2_ itself. We have not eliminated potential mechanisms involving an increase in the number of injectisomes per bacterium or an increase in the efficiency of injection per cell. However, we did not observe a significant difference in the amount of cell-associated ExoU-S142A when cells were untreated or treated with PI(4,5)P_2_ (Fig. 5B).

### Inter-relationships of PI(4,5)P_2_ in the focal adhesion, cytoskeletal structure, and plasma membrane

PI(4,5)P_2_ facilitates actin polymerization and filament assembly beneath the plasma membrane to regulate the integrity and dynamics of the sub-plasmalemmal cytoskeleton [44], [61], [64]. In addition, PI(4,5)P_2_ inhibits actin disassembly (depolymerizing and severing actin filaments) by coupling with the disassembling type of actin-binding proteins [44], [61], [65], [66]. Because actin filaments are dynamic and form both stable and labile structures, depletion of PI(4,5)P_2_ from membranes lowers adhesion energy and triggers actin depolymerization, destabilizing the cytoskeletal structure [42]. This type of phenomenon may account for the observed actin depolymerization when HeLa cells were infected with ExoU-expressing strains.

Interaction of PI(4,5)P_2_ to talin and vinculin promotes the linking of integrins to the actin cytoskeleton and the linking of the actin cytoskeleton to the membrane at the site of adhesion, respectively [44],[45],[67]. PI(4,5)P_2_ depletion from focal adhesions induces a loss of biological regulation of cell adhesion, integrity and shape, causing detachment and rounding of cells and membrane blebbing [45], [66]. Thus, degradation of PI(4,5)P_2_ by ExoU can disrupt the focal adhesion, the linkages between integrins and the actin cytoskeleton and the plasma membrane, leading to the cell detachment, cell rounding, and membrane blebbing (Figs. 2 and 6). Another possibility is that cleavage of PI(4,5)P_2_ by ExoU releases a second messenger that influences a cell signaling pathway related to the cytoskeletal and focal adhesion biology.

PI(4,5)P_2_ functions as a scaffold and recruits effectors involved in the regulation of the actin cytoskeleton [68]. There is a possibility that high affinity binding of ExoU to PI(4,5)P_2_ may compete with and replace the adaptor proteins involved in linking the actin cytoskeleton and focal adhesion to membrane bilayers. In this study, ExoU effectively competed with the PLCδ1 PH domain for PI(4,5)P_2_ binding *in vitro* (Fig 5H). However, a nontoxic form of this toxin, ExoU-S142A, demonstrated little effect on cytoskeletal structures or cell rounding, indicating that this interaction alone is likely not sufficient and PLA_2_ activity is necessary for the cytoskeletal changes, cell rounding and intoxication process.

### Use of PI(4,5)P_2_ in eukaryotic host cells by other bacterial pathogens

A number of other microbial pathogens target and use phosphoinositides as a scaffold for recruitment of effector proteins or as a tool to subvert or activate PI(4,5)P_2_-associated signaling pathways for their own advantage [68]. For example, enteropathogenic *Escherichia coli* induces the accumulation of PI(4,5)P_2_ and PI(3,4,5)P_3_ at infection sites by activating cellular phosphoinositide kinases for optimal adherence to the host cell surface [69]. Also, several bacterial virulence factors are known to act as phosphoinositide phosphatases or adaptor proteins, utilizing phosphoinositide-based cellular signaling to disrupt plasma membrane integrity [70].

An effector protein IpgD from *Shigella flexneri* is a phosphoinositide 4-phosphatase and facilitates the internalization of this bacterium into host cells [46], [71]. At *S. flexneri* entry sites, local PI(4,5)P_2_ breakdown by IpgD enzymatic activity causes remodeling of the plasma membrane and cytoskeletal structures while the product PI(5)P activates the PI3 kinase/Akt pathway [71]. Due to the regulatory role of PI(4,5)P_2_ in the adhesion of the actin cytoskeleton to the cell cortex [42], catabolism of PI(4,5)P_2_ decreases the attachment of the plasma membrane to cytoskeletal anchoring proteins, causing massive cell blebbing, actin rearrangement, and cell rounding [46], [47]. These cellular alterations facilitate internalization of *S. flexneri* into the host cell.

SopB (also known as SigD), a type III effector of *Salmonella enterica* serovar Typhimurium encoded within pathogenicity island-1 (SPI-1), possesses polyphosphoinositide phosphatase activity (reviewed in [43]). Catabolism of PI(4,5)P_2_ in the plasma membrane at the bacterial entry site enhances the invasion of host cells by coordinating with actin cytoskeleton rearrangements [59], [72], [73]. Once injected, SopB activity facilitates internalization of the bacterium into a specific intracellular compartment by hydrolysis of PI(4,5)P_2_ located on the *Salmonella-*containing vacuole membrane. This multi-functional effector is also involved in Akt activation and manipulation of the *Salmonella-*containing vacuole trafficking [73]–[76].

Another type III effector protein from *Vibrio parahaemolyticus*, VPA0450, causes autophagy and rounding of host cells, leading to cell lysis [47]. VPA0450 is an inositol polyphosphate 5-phosphatase and hydrolyzes PI(4,5)P_2_ as a main substrate. Removal of this phospholipid disrupts the cytoskeletal binding site on the inner leaflet of plasma membranes and membrane docking, leading to membrane blebbing and cell rounding during early stages of infection [47]. Extensive membrane blebbing facilitates cell lysis at later stages of infection. Although transfection of HeLa cells with *vpa0450* causes membrane blebbing, the expression of this phosphatase alone is not sufficient for cell lysis. The activities of VopQ and VopS, which destabilize the actin cytoskeleton, are necessary to achieve cell lysis [47].

Similarly, our data suggest that ExoU reduces PI(4,5)P_2_ levels in the plasma membrane by PLA_2_ activity and alters focal adhesion and actin dynamics by local detachment of the cortical cytoskeleton from the plasma membrane, leading to cell rounding. At a later stage of infection with an ExoU-expressing strain, the unbalanced and weakened cytoplasmic membrane causes membrane blebbing, which accelerates the rupture of the outer leaflet of the plasma membrane and cell lysis. Compared to the *Vibrio* VPA0450 phosphatase which requires other effectors for host cell lysis, ExoU alone is capable of inducing cell lysis through its actions that facilitate both cytoskeletal collapse and membrane blebbing using the distinctive PI(4,5)P_2_-associated PLA_2_ activity.

### Models: Modalities of the ExoU intoxication mechanism

We demonstrate that PI(4,5)P_2_ plays an important role in facilitating ExoU-mediated intoxication and cytotoxicity. In prokaryotes, ExoU is nontoxic due to a lack of enzymatic activity in the absence of its cofactor. Interestingly, when a soluble ubiquitin cofactor is co-expressed, ExoU is able to kill *E. coli*, in which PI(4,5)P_2_ is absent [28]. Also, ExoU lyses liposomes constituted with synthetic phospholipids excluding PI(4,5)P_2_ [26], [58]. These data support at least two modes in the ExoU intoxication mechanism: PI(4,5)P_2_-associated and PI(4,5)P_2_-independent.

#### 1) PI(4,5)P_2_-associated modality: Perturbation of the cytoskeletal/membrane interface, which facilitates plasma membrane injury leading to cell death

**Figure 7:**
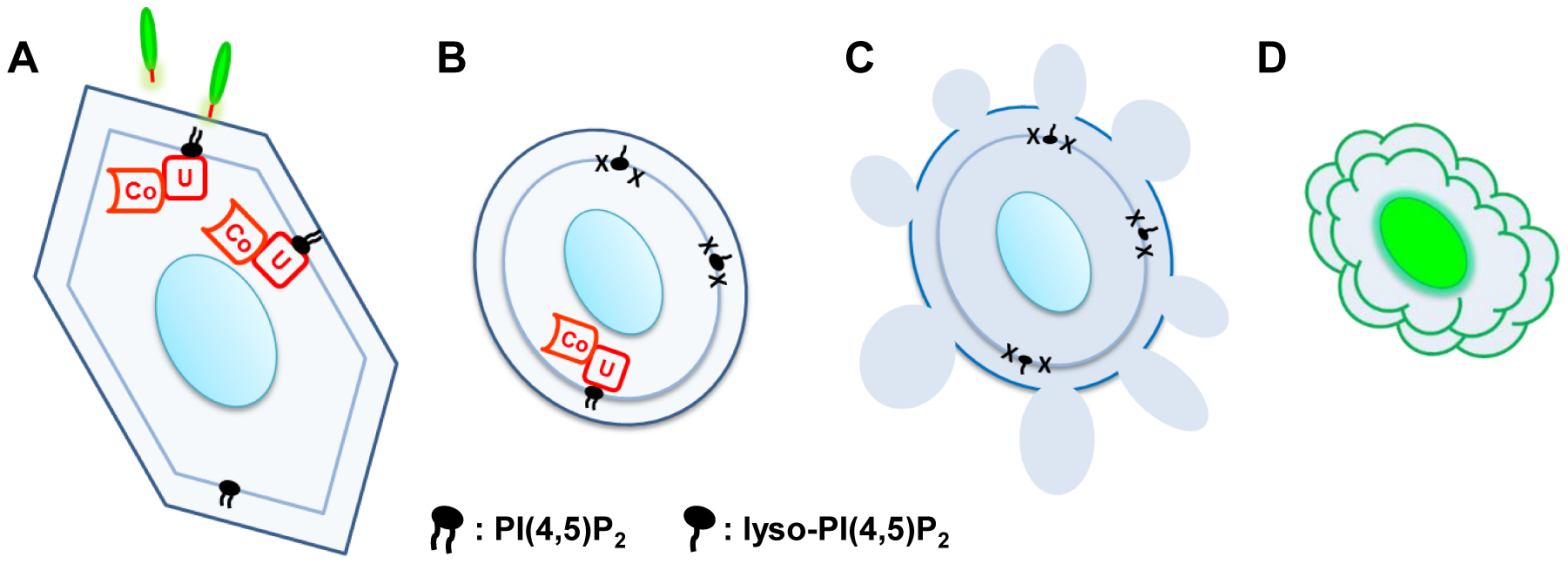
PI(4,5)P_2_-associated intoxication model: intracellularly-induced systemic death. **A**) After translocation into the host cytoplasm by T3SS, ExoU (U) is postulated to bind to PI(4,5)P_2_ residing on the inner leaflet of the plasma membrane. The PLA_2_ activity of ExoU is activated by a cofactor (Co), ubiquitin or ubiquitinated proteins. **B**) ExoU hydrolyzes PI(4,5)P_2_ and/or other phospholipid species (shown as Xs), leaving lyso-phospholipids, destabilizing the membrane. Reduction of PI(4,5)P_2_ disrupts the anchoring and interaction between the focal adhesion, cytoskeletal structure, and plasma membrane, possibly through a cell signaling pathway. Cells round due to the weakened adhesion capability and actin filament depolymerization, leading to cytoskeletal collapse. The outer leaflet of the plasma membrane is intact at this stage. **C**) At a middle stage of infection, the plasma membrane starts to bleb, facilitating further membrane damage. **D**) During a late stage of infection, the outer leaflet of the plasma membrane is compromised, allowing the influx of an impermeable nucleic acid dye and staining of the nucleus (green), consequently large molecules (e.g. LDH) are released from lysed cells.

The PI(4,5)P_2_-associated injury mode is intracellularly induced, progresses through consecutive physical destructive stages, and results in cell death (Fig 7). In the early stage of infection, ExoU preferentially binds to PI(4,5)P_2_ and disrupts the cytoplasmic side of the plasma membrane by hydrolysis of PI(4,5)P_2_ and possibly neighboring phospholipids (Fig 7A). The perturbation of PI(4,5)P_2_ lipid species weakens the association between focal adhesion proteins attached to this glycophospholipid and the plasma membranes. Also, products from PI(4,5)P_2_ hydrolysis may act as a second messenger signal. Consequently, cells peel away from their attachment anchor, lose shape and become rounded by a breakage of adhesive contacts at the edge of the cell (Fig 7B). Biological changes in this stage also include weakening of the intracellular adhesion between the cytoskeleton and plasma membrane and also disruption of the cortical cytoskeleton. This event, in turn, facilitates actin depolymerization and the collapse of cytoskeletal and subcellular structures accompanied by interrupting the actin regulatory function of PI(4,5)P_2_. During these intracellular processes, the outer leaflet of the plasma membrane is intact and cells are alive and metabolically active.

During a middle-stage of infection, plasma membrane blebbing is evident and descriptive of an uncompromised outer leaflet (Fig 7C). Blebs are created from a local disruption between the cortical cytoskeleton and the plasma membrane. Although ExoU causes cell rounding and vigorous membrane blebbing, characteristics of blebs differ from the features caused by apoptosis or other cellular phenomena (Table S1 and [44]). The size of ExoU-induced blebs is larger and ExoU also causes tubule formation (Table S1).

In the cellular environment, blebbing is generally a physical rather than a chemical process [77]. In tissue culture cells under normal biological conditions, prolonged blebbing does not compromise the barrier function of the plasma membrane [78]. ExoU-induced membrane blebbing normally continues 30 min or longer under our experimental conditions. Membrane movement becomes more vigorous over time, and the dynamic blebbing ceases upon the rupture of the outer leaflet membrane. As seen in the infection with *V. parahaemolyticus* expressing the VPA0450 phosphatase effector [47], ExoU-mediated membrane blebbing appears to facilitate membrane damage (Fig 7C and D) and subsequent cell lysis (LDH release).

Overall, the PI(4,5)P_2_-associated mode is intracellularly induced and followed by mechanical and systemic alterations of the focal adhesion, cytoskeletal structure, and plasma membrane. The end-point event in this mode is a disruption of the outer leaflet membranes and sudden membrane rupture or cell lysis, which is facilitated by membrane blebbing.

#### 2) PI(4,5)P_2_-independent modality: Direct plasma membrane injury and rupture

The direct membrane disruption/rupture mode is explicitly executed in our engineered *E.coli* strain that co-expresses ExoU and its cofactor ubiquitin [28]. This dual-expression system allows the singly-controlled expression of ExoU and soluble mono-ubiquitin. *E. coli* is killed only when both proteins are expressed simultaneously [28]. Because singly-expressed ExoU is nontoxic to bacteria, the dual-expression approach provides a selective intoxication model using bacteria as the host.

In addition to ExoU toxicity in the *E. coli* dual-expression system, catabolism of PI(4,5)P_2_-excluded synthetic liposomes by PLA_2_ activity support the model of direct membrane rupture independent of PI(4,5)P_2_. Phosphatidylinositol rarely exists in prokaryotes, except in Actinomycetes such as *Mycobacterium* and *Corynebacterium* [79], [80]. In contrast to the preferential binding to PI(4,5)P_2_ in eukaryotes, the association of ExoU to other phospholipid species was subtle in biochemical assays (Fig 5 and [36]), suggesting that transient or weak interaction is sufficient for degradation of bacterial membranes.

### Biological relevance and significance

An important characteristic of ExoU is that this toxin is unable to intoxicate from outside of the host cell *in vitro* even when a cofactor, PI(4,5)P_2_, or both are present (Table 1). Incubation of extracellularly-added rExoU or ExoU released from PA103Δ*pcrV* in the presence of a cofactor did not damage cultured cells (Table 1). In contrast, addition of honeybee PLA_2_ was easily able to damage HeLa cells (Fig 3C). In a mouse model, PA103Δ*pcrV*-infected animals show no signs of lung epithelial injury and survive as uninfected mice [53], indicating little to no toxic effects of ExoU that is released in the extracellular environment during infection. Thus, ExoU requires type III delivery to access intracellular factors and structures to damage host cells making this intoxication mechanism and its intracellular origin biologically complex.

PI(4,5)P_2_ possesses important roles in cell physiology and ExoU discriminately utilizes this phospholipid to enhance its toxicity in eukaryotic cells. The affinity of ExoU for PI(4,5)P_2_ promotes cellular injury through the alteration of the focal adhesion and cytoskeletal infrastructure, leading to cytoskeletal collapse and membrane blebbing that accelerates membrane rupture and cell lysis. This overwhelming systemic stress induces cell death in a timely manner to overcome the host membrane repair system and innate immunity, ensuring the intoxication process by this PLA_2_ toxin.

## Materials and Methods

### Bacterial strains

*P. aeruginosa* PA103 strains used in this study are shown in Table 2.

**Table 2:** *P. aeruginosa* PA103 strains used in this study.

bacterial strains	description
PA103ΔUT + pUCP-*exoU* (PA103ΔUT + *exoU*)	ExoU and ExoT knockout strain harboring a plasmid copy of *exoU*, ref [20]
PA103ΔUT + pUCP-*exoU-S142A* (PA103ΔUT + *exoU-S142A*)	ExoU and ExoT knockout strain harboring a plasmid copy of *exoU* with a point mutation at its catalytic site (S142), ref [26]
PA103ΔUT + pUCP (PA103ΔUT)	ExoU and ExoT knockout strain harboring a vector plasmid, ref [20]
PA103 *exoT::Tc* + pUCP (PA103 *exoT::Tc*)	ExoT knockout strain possessing *exoU* on the chromosome, harboring a vector plasmid, ref [20]
PA103 *exoT::Tc* + pUCP-pntp-*ngfp* (PA103 *exoT::Tc* + *gfp*)	ExoT knockout strain possessing *exoU* on the chromosome, harboring a plasmid copy of *ngfp*, in this study
PA103ΔpcrV + pUCP (PA103Δ*pcrV*)	PcrV translocator knockout strain harboring a vector plasmid, ref [53]

### Infection of human epithelial cells

HeLa (CCL-2) and A549 (CCL-185) cells were purchased from ATCC and fibroblast cells were a gift from Dr. Scott Terhune (Medical College of Wisconsin). Human epithelial cells were seeded, grown overnight in DMEM supplemented with 10% fetal bovine serum (FBS) in a tissue culture plate at 37°C with 5% CO_2_. Cells were washed with HBSS twice and bacterial cells suspended in serum-free DMEM were added for infection at an MOI of 2.5 unless indicated. Occasionally, an MOI of 1.25 or 5 was used depending on the timing of an intoxication phase studied or the sensitivity of an assay used. For example, the data shown in Figure 4C, an MOI of 1.25 was used to detect the increased toxicity by PI(4,5)P addition with the propidium iodide assay, which detects the early stage of membrane damage. For Figure 5B, an MOI of 5 was used to detect the low amounts of ExoU-S142A translocated into HeLa cells via type III system.

### Lactate dehydrogenase (LDH) release assay to measure cytotoxicity

Cells (1×10^5^) were seeded in a 24-well tissue culture plate or 4×10^5^ cells in a 6-well plate, grown overnight prior to infection. To follow the release of LDH, aliquots of the tissue culture medium were subjected to centrifugation to exclude floating cells and debris. The supernatant was diluted in serum-free DMEM (4-fold higher dilution for the 6-well format) and LDH activity was measured using the CytoTox 96H Non-Radioactive Cytotoxicity Assay (Promega) according to manufacturer's instructions. LDH activity was detected by using a SpectraMax M5 microplate reader (Molecular Devices). In addition to the absorbance values in graphs, percent cytotoxicity (shown in the text) was calculated using a supernatant from cells exposed to lysis buffer as the maximum reference and uninfected samples as the baseline. Assays were performed at least 3 times as independent experiments and the error bars indicate standard deviations (SD) from the mean.

### Flow cytometric analysis to quantify the population of ExoU-intoxicated cells or cells containing translocated ExoU-S142A

HeLa and A549 cells (1.5×10^6^) were seeded in a 60 mm culture dish. After overnight incubation, cells were infected with PA103ΔUT + *exoU* or *exoU-S142A*. For quantification of intoxicated cells, infected cells were stained with propidium iodide (1 µg/ml, Sigma), rinsed, and trypsinized. The trypsin enzymatic activity was quenched by DMEM containing 10% fetal bovine serum. Cells were collected by centrifugation and suspended in PBS. The same volume of 4% para-formaldehyde (Electron Microscopy Sciences) was added for fixation at room temperature for 15 min and then washed with PBS before analysis.

For immunofluorescence-based analysis, fixed cells were blocked with 3% bovine serum albumin (BSA) in PBS, permeabilized with 0.1% Triton X-100 in 3% BSA/PBS for 2 min on ice, washed with 3% BSA/PBS. Permeabilized cells were labeled with the anti-ExoU monoclonal antibody (mAb) U7.15 and Alexa Fluor 488-conjugated anti-mouse IgG Ab (Molecular Probes).

For flow cytometric analysis using the Guava easyCyte 6HT (Millipore), samples were diluted with PBS to contain less than 500 cells/µl for 5,000 events per run. The total cell numbers were evaluated with forward scatter. Propidium iodide-stained and immunolabeled cells were detected by using a specific laser for fluorescence. Guava ExpressPro was used for analysis. Samples were prepared in duplicate from at least two independent experiments for statistical analyses with SD.

### Triton-based fractionation of infected cells and Western blot analysis to evaluate the amount of translocated ExoU-S142A

HeLa cells (1.8×10^6^) were seeded in a 60-mm dish overnight and infected with PA103ΔUT + *exoU-S142A* at an MOI of 5. Infected cells were washed and lysed with 0.05% Triton X-100/10 mM EDTA in PBS. Membrane fractions were collected by ultracentrifugation at 80,000×*g* for 30 min. Proteins were separated by SDS-PAGE and lanes were normalized based on volumes. Separated proteins were transferred to nitrocellulose membranes and probed with the anti-ExoU mAb U29F8 (1∶20,000) followed by a horseradish-peroxidase conjugated anti-mouse IgG Ab (1∶10,000, Roche). SuperSignal West Pico Chemiluminescent Substrate (Thermo Scientific) was used for signal detection. The signals from chemiluminescent analyses were quantified by using an AlphaImager AIC (Alpha Innotech) for densitometry.

### Immunofluorescence microscopy

HeLa cells (3.6×10^5^) were grown on fibronectin-coated coverslips in a 35-mm culture dish overnight prior to infection. Infected cells were fixed with 2% para-formaldehyde for 15 min and washed with PBS five times. Prior to immunostaining, fixed cells were permeabilized with 0.1% Triton X-100 in 10% FBS/PBS for 10 min and blocked in 10% FBS/PBS to reduce nonspecific binding of antibodies. Primary antibodies were diluted in 10% FBS/PBS and incubated with the permeabilized cells for 60 min. After rinsing the primary antibody with 10% FBS/PBS, cells were incubated with Alexa Fluor-labeled secondary antibodies for 60 min. The samples were washed thoroughly with 10% FBS/PBS and then with PBS prior to mounting on a slide glass using ProLong Gold (Invitrogen).

Antibodies used for immunofluorescence microscopy are: anti-alpha-tubulin mouse mAb (1∶2,000, Sigma) and anti-*P. aeruginosa* LPS rabbit polyclonal Ab (1∶400,000, a kind gift from Dr. Gerald Pier, Channing Laboratory, Harvard University). For these primary antibodies, an Alexa Fluor 488 or 568 conjugated secondary Ab was used at 1∶2,000 dilution (Molecular Probes). For the anti-zyxin mouse mAb (1∶1,000, Millipore), an anti-mouse IgM (µ chain)-Alexa Fluor 647 secondary Ab (1∶1,000, Molecular Probes) was used.

F-actin was stained with Alexa Fluor 568-conjugated phalloidin (1∶600 or ∼11 nM) while G-actin was stained with 0.3 µM Alexa Fluor 488-conjugated DNase I (both from Molecular Probes).

Fluorescence microscopy images were acquired by a Nikon Eclipse Ti-U inverted microscope equipped with a CoolSNAP ES2 CCD camera (Photometrics) and a multifluorescent Sedat Quad ET filter set (multichroic splitter, Chroma). NIS-Elements software (Nikon) was used for image acquisition and analysis. The objective lens used were 20× Plan Apo (N.A. 0.75) and 60× Plan Apo VC (N.A. 1.40 oil) from Nikon.

### Live cell staining and time-lapse fluorescence microscopy

HeLa cells (4×10^5^) were seeded in a 35 mm dish and grown overnight prior to infection or staining. For the time-lapse imaging of live cells, DMEM (free of phenol red or serum) was diluted to 34% with HBSS and buffered with 15 mM HEPES. During image acquisition, the cells were kept at 36°C by a temperature-controlled platform (Warner Instruments).

ExoU cytotoxicity was analyzed when cells were infected with PA103 *exoT::Tc*, a strain expressing only ExoU from a single copy chromosomal gene. For comparison of ExoU phenotypes to phenotypes that may occur due to expression of a nontoxic allele, cells were infected with PA103ΔUT + *exoU* and PA103ΔUT + *exoU-S142A* strains. Reagents used to induce other types of cell death are 3 µM gliotoxin (EMD Calbiochem), 100 µl of 0.1% Triton X-100 (Sigma), 675 pmol PLA_2_ from honey bee (*Apis mellifera*, 14.5 kDa, Biomol), and 20 µM lyso-PC (Avanti Polar Lipids).

Fluorescent dyes used for live cell imaging were CellMask Orange plasma membrane stain (1 µg/ml), impermeable SYTOX green nucleic acid stain (75 nM) or SYTOX blue (150 nM), and permeable Hoechst 33342 dye (5 µg/ml), all purchased from Molecular Probes.

For temporal analysis of ExoU-mediated biological changes in HeLa cells, subcellular structures were labeled via intracellular-targeted GFP using a baculovirus system from Molecular Probes (CellLight BacMam). Actin structures and focal adhesions in HeLa cells were labeled with GFP-fused actin and talin-specific signal peptide-fused with GFP, respectively. For the transduction procedure (26 h) to visualize the expression of actin-GFP and talin-targeting peptide-GFP, 50 and 37.5 particles per cell were used, respectively. After confirming the GFP expression by microscopy, cells were washed and infected with PA103 *exoT::Tc*.

For time-lapse microscopy, images were acquired by using the Nikon Eclipse Ti-U microscope, as described in the immunofluorescence microscopy section, with an objective lens 40× Plan Fluor (N.A. 0.60, Nikon).

### Effect of PI(4,5)P_2_ on ExoU-mediated cytotoxicity in HeLa cells

HeLa cells were grown in a 24-well plate (see LDH release assay), pre-incubated with 2.5 nmol PI(4,5)P_2_ (Echelon) or POPC (Avanti Polar Lipids) or without additional phospholipids for 1 h, and infected with PA103ΔUT + *exoU* or *exoU-S142A*. Cytotoxicity was analyzed by either a cell retention assay using a crystal violet stain (Hardy diagnostics) according to manufacturer's instructions, staining with propidium iodide (0.25 µg/ml), or an LDH release assay as described.

### Colony forming unit (CFU) assay to evaluate bacterial growth during HeLa infection

HeLa cells (9.8×10^4^) were seeded in a 24-well cell culture plate, grown overnight. Cells were preincubated with or without 2.5 nmol phospholipids for 1 h, and infected at an MOI of 5. At 4 hpi, the cell culture medium containing bacterial cells was diluted by 10-fold serial dilutions, plated on Vogel-Bonner minimal medium agar plates containing 400 µg/ml carbenicillin. After overnight incubation, bacterial colonies on each plate were counted for evaluation. The data represents the mean with SD from triplicates of 2 independent experiments.

### Thin-layer chromatography (TLC) to analyze phospholipase activity on PI(4,5)P_2_

Hydrolysis of PI(4,5)P_2_ by rExoU and other phospholipases was examined by TLC analysis using BODIPY-FL-PI(4,5)P_2_, C_16_ (Echelon) as a substrate. The fluorescent substrate (50 to 75 pmol) was incubated with 13.5 pmol of PLA_2_, PI-PLC, or rExoU (13.5 pmol or 6.76 pmol) with 13.5 pmol of the cofactor penta-ubiquitin at room temperature for 16 h in dark. The hydrolyzed products were spotted on a silica gel plate, dried at 110°C for 15 min, and then eluted with a 1-propanol-based solvent system consisting of 1-propanol/2 M acetic acid (65∶35). The samples were also eluted with another chloroform-based system, chloroform/acetone/methanol/acetic acid/water (80∶30∶26∶24∶14) for confirmation of the results (not shown). Eluted BODIPY signals were detected by Typhoon FLA7000, quantified with ImageQuant TL (both GE Healthcare). rExoU or the cofactor alone did not possess detectable phospholipase activity (not shown).

### Mechanical lysis and differential centrifugation-based fractionation of infected cells

The localization of ExoU-S142A in HeLa cells was analyzed after infection in the presence or absence of the synthetic phospholipids. HeLa cells (1.4×10^6^) were seeded in a 60 mm culture dish, grown overnight, and incubated with 7.4 nmol phospholipid in 2 ml serum-free DMEM for 30 min. The bacterial inoculum was then added for an MOI of 5. After 4.5 h incubation, cells were washed, scraped into lysis buffer containing 250 mM sucrose, 3 mM imidazole, protease inhibitor cocktail (Complete Mini, Roche), and lysed by 6 passages through a 21G1 needle, followed by an additional 6 passages through a 25G1½ needle. Unbroken HeLa cells, nuclei, cytoskeleton, and bacteria were pelleted by centrifugation at 3,000×*g* for 15 min. The supernatant was subjected to ultracentrifugation at 80,000×*g* for 30 min to isolate the membrane fraction from the cytoplasmic fraction.

Fractions were subjected to SDS-PAGE and the cellular location of ExoU-S142A was analyzed by immunoblotting as described. To achieve a linear detection range, lanes containing membrane samples were loaded with volumes that were 4-fold higher as compared to soluble fractions. The detected signals were analyzed by densitometry using ImageQuant TL (GE Healthcare).

### ELISA-based solid-phase binding assay to quantify the affinity of rExoU to phospholipids

Phospholipids at the indicated amounts were immobilized in a 96-well ELISA polystyrene plate (BD Falcon) by incubation at room temperature for 1 h and dried under vacuum. Excess phospholipids were washed with Tris-buffered saline (TBS) and each well was blocked with 1% BSA in TBS for 2 h. rExoU (13.5 pmol) was added and incubated for 2 h to allow binding. Unbound rExoU was washed away with TBS prior to the incubation with the anti-ExoU mAb U29F8 (1∶1,000). After 1 h incubation, wells were washed and incubated with a horseradish peroxidase-conjugated anti-mouse IgG Ab (1∶1,000, Roche) for 1 h. After another set of washes, the binding of rExoU to phospholipids was measured by assessing peroxidase activity with the substrate ABTS with H_2_O_2_ (22 µg ABTS/100 µl in the presence of H_2_O_2_ [1∶1,000 dilution of 30% H_2_O_2_] in 0.1 M citrate-phosphate buffer, pH 4). Peroxidase activity was detected at absorbance 405 nm by using a SpectraMax M5 microplate reader (Molecular Devices).

To determine the Kd of rExoU binding to PI(4,5)P_2_, the ELISA-based binding assay was slightly modified. A constant concentration of PI(4,5)P_2_ (200 or 400 pmol) was immobilized on a plate and 6.25 nM to 1600 nM of rExoU (2-fold dilutions) were bound. Each concentration of rExoU was also incubated in the blocked wells without phospholipids for nonspecific binding controls. Kd and Bmax were analyzed using a nonlinear regression, one site binding saturation fit model in Prism 5 (GraphPad Software). Nonspecific binding and background values were subtracted prior to analysis. The mean ± SEM from at least three independent experiments are reported.

To determine the Kd of the PLCδ1-PH domain to PI(4,5)P_2_ (200 pmol), a range of 6.25 nM to 400 nM of the PLCδ1-PH peptide (PI(4,5)P_2_ binding protein, Cayman Chemical) were used. To evaluate the competition between rExoU and the PLCδ1-PH peptide for PI(4,5)P_2_ binding, 6.25 to 800 nM of rExoU was mixed with 50 nM of the PLCδ1-PH peptide. The IC_50_ of rExoU to displace 50% of PLCδ1-PH binding was determined using a nonlinear regression, one site fit log IC_50_ model in Prism 5. The mean ± SEM from at least three independent experiments is reported.

### Detection of phospholipid binding of rExoU by fluorescence microscopy

A constant amount (1600 pmol) of phospholipids was immobilized on a polystyrene plate or a glass slide. The binding of rExoU (13.5 pmol) was detected by fluorescence microscopy using an anti-ExoU mAb and an Alexa Fluor 488-conjugated secondary Ab. Incubation and washing conditions were the same as the ELISA-based binding assay. The signal of rExoU binding to each phospholipid was detected by fluorescence microscopy using a 4× objective lens. Images were acquired with the same exposure time for all samples.

### Statistical analysis

Statistical comparisons were analyzed by *t*-test using SigmaPlot version 11.0.

## Supporting Information

Table S1 Comparison of the size of ExoU-mediated blebs to apoptotic blebs. *average values in µm.
(DOCX)

Movie S1 Cell rounding and plasma membrane blebbing when HeLa cells were infected with a *P. aeruginosa* strain expressing both ExoU and GFP at an MOI of 2.5 (corresponding to Fig 2B). The CellMask plasma membrane stain (red) and permeant Hoechst 33342 (blue) were added 20 min before start imaging at 4.5 hpi. The images were acquired by time-lapse microscopy for 20 min with 20 sec interval.
(AVI)

Movie S2 Cell rounding and dynamic membrane blebbing prior to the rupture of plasma membranes indicated by the influx of SYTOX green (corresponding to Fig 2C). Cells were infected with a strain expressing ExoU at an MOI of 5 for 3.5 h. Cells were also stained with CellMask plasma membrane stain (red) and Hoechst 33342 (blue). The images were acquired by time-lapse microscopy for 1 h with 1 min interval.
(AVI)

Movie S3 Gliotoxin-induced apoptotic nuclear shrinkage and membrane blebbing (corresponding to Fig 3A). No influx of SYTOX green was observed during image analysis. Cells were also stained with CellMask plasma membrane stain (red), Hoechst 33342 (blue). The images were acquired by time-lapse microscopy for 15 min with 30 sec interval.
(AVI)

Movie S4 Surfactant-induced membrane damage resulted in cell death (corresponding to Fig 3B). None of cell rounding, nuclear shrinkage, or membrane blebbing phenotype was observed. SYTOX green (impermeant), CellMask plasma membrane stain (red) and Hoechst 33342 (blue) were used for visualization. The images were acquired by time-lapse microscopy for 10 min with 15 sec interval.
(AVI)

Movie S5 Lyso-phospholipid-induced cell death. The addition of 20 µM lyso-PC, a product of PLA_2_ enzymatic activity, intoxicated cells without cell rounding, nuclear shrinkage, or the membrane blebbing phenotype. The nucleus and cell swelled upon cell lysis, which is characteristic of necrosis. Cells were visualized by staining with SYTOX green, CellMask plasma membrane stain (red), Hoechst 33342 (blue). The images were acquired by time-lapse microscopy for 40 min with 30 sec interval.
(AVI)

Movie S6 Honeybee PLA_2_-induced cell death (corresponding to Fig 3C). Cells were stained with CellMask plasma membrane stain (red), Hoechst 33342 (blue), and SYTOX green. The images were acquired by time-lapse microscopy for 35 min with 1 min interval.
(AVI)

Movie S7 Depolymerization of actin filaments when HeLa cells were infected with a strain expressing ExoU (corresponding to Fig 6C). Cells were labeled with CellLight actin-GFP, CellMask plasma membrane stain (red), Hoechst 33342 (blue). The images were acquired by time-lapse microscopy for 30 min with 30 sec interval.
(AVI)

Movie S8 Effect of ExoU on focal adhesion in infected HeLa cells (corresponding to Fig 6D). Cells were visualized with CellLight Talin-targeted GFP, CellMask plasma membrane stain (red), Hoechst 33342 (blue). The images were acquired by time-lapse microscopy for 40 min with 30 sec interval.
(MOV)
