# Supplementary material for: Intoxication of Host Cells by the T3SS Phospholipase ExoU: PI(4,5)P2-Associated, Cytoskeletal Collapse and Late Phase Membrane Blebbing
Source: PLoS One. 2014 Jul 25;9(7):e103127. doi: 10.1371/journal.pone.0103127 (PMC4111512; doi:10.1371/journal.pone.0103127)
Supplement: Table S1 — Comparison of the size of ExoU-mediated blebs to apoptotic blebs. *average values in µm. (DOCX) [file pone.0103127.s001.docx]

**Table S1.** Comparison of the size of ExoU-mediated blebs to apoptotic blebs.

|  | **Diameter*** | **SD** |  |
| --- | --- | --- | --- |
| **apoptotic blebs** | 2.02 | 0.87 | N = 30 |
| **ExoU blebs** | 7.82 | 2.06 | N = 49 |
|  | **Length*** |  |  |
| **ExoU tubules** | 20.18 | 6.55 | N = 14 |

*average values in µm.
